# Successful Elimination of Endemic Rubella in the WHO European Region. Is It Proper to Remove the Recommendation for Preconceptional Immunization?

**DOI:** 10.3390/ijerph21070957

**Published:** 2024-07-22

**Authors:** Anna Franca Cavaliere, Marco Parasiliti, Rita Franco, Vitalba Gallitelli, Federica Perelli, Amelia Spanò, Barbara Pallone, Maria Grazia Serafini, Fabrizio Signore, Georgios Eleftheriou, Giovanni Scambia, Antonio Lanzone, Annalisa Vidiri

**Affiliations:** 1 Department of Gynecology and Obstetrics, Ospedale Isola Tiberina-Gemelli Isola, 00186 Rome, Italy; annafranca.cavaliere@fbf-isola.it (A.F.C.); rita.franco@fbf-isola.it (R.F.); vitalba.gallitelli.fw@fbf-isola.it (V.G.); barbara.pallone@fbf-isola.it (B.P.); annalisavidiri@gmail.com (A.V.); 2 Division of Gynecology and Obstetrics, Santa Maria Annunziata Hospital, USL Toscana Centro, 50012 Florence, Italy; federica.perelli@uslcentro.toscana.it; 3 Department of Science of Woman, Child and Public Health, Fondazione Policlinico Universitario A. Gemelli IRCCS, Università Cattolica del Sacro Cuore, 00168 Rome, Italy; ameliaspano94@gmail.com (A.S.); giovanni.scambia@policlinicogemelli.it (G.S.); antonio.lanzone@policlinicogemelli.it (A.L.); 4 Department of Gynecology and Obstetrics, Sant’Eugenio Hospital, ASL Roma 2, 00144 Rome, Italy; mariagrazia.serafini@aslroma2.it (M.G.S.); fabrizio.signore@aslroma2.it (F.S.); 5 Poison Control Center, Hospital Papa Giovanni XXIII, 24127 Bergamo, Italy; jorgos_2002@yahoo.com

**Keywords:** congenital rubella syndrome, rubella seroprevalence, preconceptional vaccination, IgG

## Abstract

Background: Rubella is a contagious viral infection that has garnered significant attention in the field of public health due to its potential consequences, especially during pregnancy. In recent decades, it has been recommended that non-immune women receive immunization during the preconceptional and/or postpartum periods. The goal of this strategy is to prevent primary rubella infection in order to protect pregnant women against congenital rubella syndrome. In November 2022, the WHO’s Regional Verification Commission declared the elimination of rubella infection in Italy. In recent years, the main migration flows to Italy have originated from regions where rubella has not yet been eliminated and where no program is in place to achieve this goal. Objective: The aim of this study was to retrospectively assess rubella immunity in pregnant women who have attended three delivery centers in Rome over the past three years, from January 2021 to May 2023. Methods: Data about the rubella serological status of 7937 non-consecutive pregnant women were collected. Univariate analysis was performed to verify any difference between the study groups in terms of age distribution. Results: Anti-rubella IgG antibodies were found in 7224 (91%) women while 713 (9%) were susceptible to rubella (IgG negative), without differences in terms of immunity rate between Italian and non-Italian women. Age analysis showed a statistically significant older age of immune women than receptive women and of Italian immune women than non-Italian immune women. Conclusions: The National Plan for the Elimination of Measles and Congenital Rubella aimed to achieve a percentage of susceptible women of childbearing age below 5%. These data indicate the relevance of maintaining the recommendation for preconceptional rubella vaccination in Italy.

## 1. Introduction

Rubella is a contagious viral infection caused by the rubella virus, an RNA-virus belonging to the family *Matonaviridae*, genus *Rubivirus*, and humans are the only reservoir for rubella infection [1]. Rubella infection has garnered significant attention in the field of public health due to its potential consequences, especially during pregnancy [2]. While rubella infection is generally manageable in most individuals and sometimes may proceed asymptomatically, the real concern arises when the virus affects pregnant women. Rubella infection in pregnant women, particularly if contracted during the first trimester, can result in congenital rubella syndrome (CRS), the clinical manifestations of which include fetal growth restriction, cardiac defects, congenital cataract, sensorineural hearing loss and bone lesions [3].

The virus is transmitted by direct droplet contact from nasopharyngeal secretions, replicates in the lymph tissue of the upper respiratory tract, and spreads hematogenously. Congenital infection occurs when maternal viremia allows hematogenous spread of the virus across the placenta. Acquired rubella is generally a mild, self-limited disease associated with a characteristic exanthem [4]. Though asymptomatic in 25 to 50 percent of cases, affected individuals may experience mild, prodromal symptoms consisting of low-grade fever, conjunctivitis, coryza, sore throat, cough, and occasionally headache and malaise. These symptoms usually last one to five days before the onset of the rash. Rubella may also be associated with generalized, tender lymphadenopathy, particularly involving suboccipital, postauricular, and cervical nodes, which often becomes pronounced during the rash. The typical rash of rubella is an erythematous maculopapular eruption, which may be mildly pruritic and evolves into pinpoint papules similar to scarlet fever. The rash characteristically begins on the face and spreads to the trunk and extremities within hours. It lasts approximately one to three days and resolves first from the face and then from the body [4].

When a woman contracts rubella just before conception or within the first 8–10 weeks of pregnancy, it can result in fetal defects in up to 90% of cases, including miscarriage or stillbirth. Maternal–fetal transmission occurs via hematogenous spread and varies with gestational age. There is considerable pathologic evidence that suggests that the rubella virus spreads through the vascular system of the developing fetus after infecting the placenta. The resulting defects stem from cytopathic damage to blood vessels and ischemia in affected organs [5]. The likelihood of birth defects decreases if the infection occurs later in pregnancy, and fetal abnormalities are rarely linked to maternal rubella after the 16th week, though sensorineural hearing loss can still occur if the infection happens as late as that. This syndrome, which can have severe consequences for pregnant women, is the only one preventable by vaccination [6].

Over the past 10 years, significant efforts have been made to reduce the incidence of rubella infections worldwide. In the European region, between 2005 to 2019, the annual incidence of rubella dropped from 234.9 per 1 million people (206,359 cases) in 2005 to 0.67 per 1 million people (620 cases) in 2019. In 2019, Poland had the highest incidence, with 7.7 cases per 1 million people (292 cases), followed by Ukraine with 3.1 cases per 1 million people (138 cases). Cases of congenital rubella syndrome (CRS) were reduced by 50%, from 16 cases in 2005 to 8 cases in 2019 [7].

The rubella vaccine is usually given as part of a measles–mumps–rubella vaccination (MMR). Because of the presence of live attenuated virus, MMR is strongly contraindicated during pregnancy and rubella immunization is appropriately recommended before the conception or in postpartum period in non-immune women. A sufficiently adequate wait time prior to conception after MMR vaccine administration corresponds to 4 weeks [8]. However inadvertent administration of the MMR vaccine early in pregnancy is it not generally related to teratogenic effects: as noted in a 2014 review by Keller-Stanislawski et al., among over 3500 susceptible women who were inadvertently vaccinated against rubella just before or during the early stages of pregnancy, no cases of congenital rubella syndrome (CRS) were reported. It has been shown that the vaccine virus can be transmitted vertically to the fetus without causing any clinical symptoms [9]. Thus, pregnancy termination is not recommended for individuals who are vaccinated during pregnancy (or become pregnant soon after vaccination) [10]. Moreover, when the vaccination occurs during the post-partum period, breastfeeding is not contraindicated.

Despite increasing scientific evidence supporting the benefits and safety of immunization before, during, and after pregnancy, vaccine hesitancy persists because many people perceive vaccines to be unnecessary and unsafe [11].

Various approaches have been taken to persuade patients who refuse vaccination during preconception period or pregnancy. It has been demonstrated that one of the most effective methods is recommendation from the attending gynecologist [12]. The most effective communication strategy has proven to be explaining to the patient that not getting vaccinated seriously endangers the fetus or newborn child. Furthermore, emphasizing the importance of vaccination has been shown to be effective. In one study, 20% of pregnant people who received the influenza vaccine had previously refused it [13].

According to the recommendations of the World Health Organization (WHO), several countries have endeavored to establish vaccination programs, surveillance and notification systems for rubella infection and CRS [14]. Considering that a single dose of rubella-containing vaccine (RCV) can confer lifelong immunity, the WHO formulated a Global Vaccine Action Plan 2011–2020 (GVAP) [3]. This plan included a target to achieve elimination of rubella in at least five of the six WHO regions by 2020 [14]. Elimination of rubella is defined as the absence of endemic transmission in a defined geographical region or country for at least 36 months, documented by a well-performing surveillance system [15]. In contrast to eradication, ongoing measures are necessary to prevent the re-establishment of transmission [14]. The WHO recommends that countries that introduce RCV achieve and maintain a minimum coverage of at least 80%, with at least 1 dose of RCV delivered through routine services or campaigns.

Until the disease is eliminated in all countries of the world, it will be necessary to maintain high vaccination coverage, and further strengthen the surveillance and investigation of reported cases, ensuring a rapid response to any imported case. Currently, only two WHO regions in the world are yet to achieve rubella elimination: the African region and the Eastern Mediterranean region [14]. In November 2022, the WHO’s Regional Verification Commission declared the elimination of rubella infection in Italy, making it no longer endemic [16]. In Italy, numerous efforts have been made to achieve rubella elimination, including mandatory vaccination for newborns since 2017. The National Plan for the Elimination of Measles and Congenital Rubella for the period 2010–2015, following the guidelines of the WHO’s European Regional Office, aimed to achieve a two-dose vaccine coverage of 95% and a percentage of susceptible women of childbearing age below 5% [17].

In recent years, the main migration flows to Italy have originated from regions where rubella has not yet been eliminated and where no program is in place to achieve this goal [14]. These flows have been particularly noted from Africa and the Middle East due to conflicts, political instability, and challenging economic conditions. In a recent study by Zenner et al., the implementation of the electronic Personal Health Record System, a health information system that records health data for newly arriving migrants, was examined in southern Europe. The analysis of these data highlighted the presence of a predominantly healthy population, but with a significant prevalence of both acute and chronic infectious diseases. Specifically, HIV, tuberculosis, and infectious hepatitis were more frequent among the older age group. This frequency could be attributed to the prevalence rates in the migrants’ countries of origin and the adverse conditions encountered during their migration journey [18].

Millions of Syrian refugees have fled their country, seeking safety in neighboring countries such as Lebanon, Jordan, or in Europe. Ten percent of these have reached Europe in recent years [19]. These migratory flows have the potential to reintroduce diseases into host countries where their incidence had significantly declined over time. These diseases include, for example, hepatitis A, which represents an emblematic case. The hepatitis A vaccine is safe and effective in preventing acute infection cases, providing immunity for at least 15 years once administered. In Europe, most countries do not include hepatitis A in their vaccination programs, unlike Middle Eastern states like Lebanon and Jordan, which are considered high–intermediate endemic countries for this infection [20]. Paradoxically, therefore, the migration of these people could be riskier in Europe than in neighboring countries, as Europe is a low-endemic region, consequently having a large number of susceptible individuals.

The increase in cases of infectious diseases related to migration flows has already been highlighted in Middle Eastern countries such as Lebanon [21]. Following the Syrian refugee crisis and the lack of clean water in this region, a group of Lebanese researchers demonstrated the high prevalence of Entamoeba histolytica infection in children below one year old, as the cause of pediatric gastroenteritis. E. histolytica is usually transmitted via contaminated food and water, meaning that young infants should be less likely to frequently develop intestinal amebiasis. However, contaminated water used for milk preparation and hygiene can be ingested by infants during bathing or face washing. This fact highlights how unsanitary water supplies and poor hygiene conditions can affect the prevalence of E. histolytica infection in infants [21].

In 2022, approximately 20.0% of births concerned mothers of non-Italian citizenship. The most represented geographical areas of origin were Africa (28.7%) and the European Union (19.6%). Mothers of Asian and South American origin make up 19.3% and 7.9% of foreign mothers, respectively [22].

A seroepidemiologic study, conducted from 1 January 2008 to June 30 2009 on 489 immigrant women who were resident in Messina and aged between 18 and 45 years, showed an overall rate of seropositivity to rubella of 82.2%. This level of susceptibility to rubella virus infection in fertile immigrant women was still too high to meet the target of the WHO’s European Regional Office [23].

Regarding the vaccination coverage rate of pregnant women, these data are not available through the official channels of the Italian National Institute of Health.

The aim of this study is to retrospectively assess rubella immunity in pregnant women who have attended three delivery centers in Rome over the past three years. The objective is to determine how many women are seronegative to rubella and are at risk of contracting the infection during pregnancy, thereby risking CRS for their newborns.

## 2. Materials and Methods

Data about the rubella serological status of 7937 non-consecutive pregnant women who delivered at three different hospitals in Rome, Italy, were collected from Isola Tiberina– Gemelli Isola Hospital, Fondazione Policlinico Universitario A. Gemelli IRCCS, and Sant’Eugenio Hospital. We have included only patients who delivered between January 2021 and May 2023 with a complete rubella test (IgM and IgG) performed during pregnancy or at the time of admission in hospital and considered only rubella tests that had been analyzed with the same method (Abbott Molecular Diagnostics, Des Plaines, USA, chemilumiscence, CLIA). The results were evaluated in terms of IgG positive (≥10 UI/mL), negative (≤4.9 UI/mL) and borderline (5–9.9 UI/mL); IgM positive (≥1.6 index), negative (<1.2 index) and borderline (1.2–1.59 index). All of the results with IgG and/or IgM borderline were excluded in order to categorize pregnant women as either receptive (both IgG and IgM negative) or immune (IgG positive—IgM negative) to rubella, avoiding non-interpretable rubella test results.

Statistical analysis. Univariate analysis was performed to verify any difference between the study groups in terms of age distribution. Univariate analysis included the Student’s t test when appropriate for continuous variables. All statistical tests were two sided, and differences were considered significant at *p* < 0.05. Statistical analysis was performed by SPSS statistical software 29.0 (SPSS, Chicago, IL, USA).

All patients admitted to the hospital signed an informed consent that allows personal data to be used in anonymous form for clinical and scientific purposes.

## 3. Results

Data about age, parity and geographical origin (Italian/not Italian) of the women enrolled were registered. We report the clinical and demographic characteristics of the sample in Table 1 and the clinical and demographic characteristics of the Italian and non-Italian populations in Table 2. Data are reported expressing the numerical value and the percentage on the sample, while the age is reported as the mean age expressed in years.

**Figure 1:**
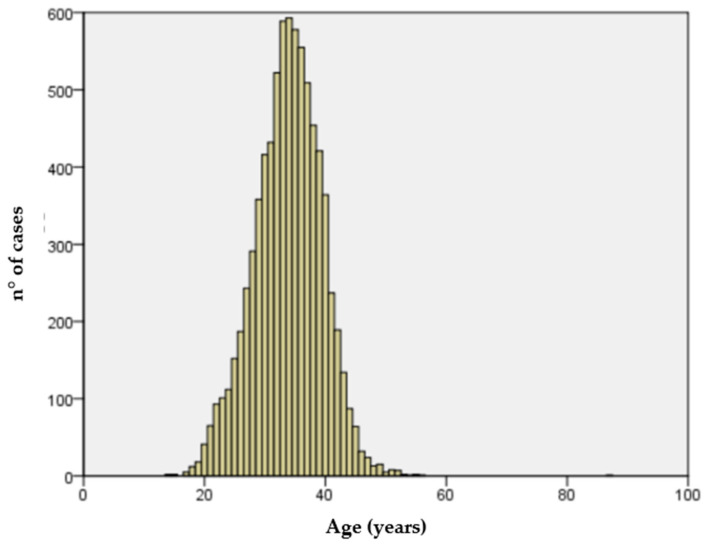
Age distribution of the sample.

We collected 7937 non-consecutive cases. The age distribution of the sample examined varies from a minimum of 14 to a maximum of 56 years old, with a mean age of 33.8 years old (Figure 1). Anti-rubella IgG antibodies were found in 7224 (91%) women while 713 (9%) were susceptible to rubella (IgG negative). No IgM-positive cases were documented.

The age analysis showed a statistically significant older age of immune women than receptive women (mean age 33.96 ± 5.6 versus 32.69 ± 5.4 years old, *p* < 0.05, Table 3).

A subgroup analysis for the 7224 immune women was performed and showed a statistically significant older age of Italian immune women than non-Italian immune women (mean age 34.36 ± 5.4 versus 32.08 ± 5.9 years old, *p* < 0.05, Table 4).

Considering rubella immune women, 3822 (52.9%) were nulliparous, 2673 (37%) had 1 child and 729 (10.1%) had at least 2 children; the mean age was 34 years old (14–56). Considering women who were found to be receptive to rubella, 420 (58.9%) were nulliparous, 216 (30.3%) had 1 child and 77 (10.8%) had at least 2 children; the mean age was 32.7 years old (17–46). Considering nationality, 6512 (82%) were Italian and 1425 (18%) were non-Italian. Among Italian population, 5938 (91.2%) were immune and 574 (8.8%) were susceptible to rubella, while among the non-Italian population, 1286 (90.2%) were immune and 139 (9.8%) were found to be susceptible to rubella. The mean age of the Italian immune population was 34.36 years old (14–56); 3325 (56%) were nulliparous, 2169 (36.5%) had 1 child and 444 (7.5%) had at least 2 children. The mean age of the Italian receptive population was 33.2 years old (20–46); 364 (63.4%) were nulliparous, 165 (28.8%) had 1 child and 45 (7.8%) had at least 2 children. The mean age of the non-Italian immune population was 32.08 years old (15–55); 487 (37.9%) were nulliparous, 508 (39.5%) had 1 child and 291 (22.6%) had at least 2 children. The mean age of the non-Italian receptive population was 30.5 years old (17–38); 56 (40.3%) were nulliparous, 51 (36.7%) had 1 child and 32 (23%) had at least 2 children.

## 4. Discussion

The data observed in our examined sample showed a percentage of pregnant women with immunity in line with national data for the general population, indicating immunity rates of 91.2% in Italian women and 90.2% in non-Italian women. No differences in terms of immunity rate were observed between Italian and non-Italian women, in contrast with previous data [23]. Approximately 9% of pregnant women in both groups were therefore susceptible to rubella infection during pregnancy, these data are in line with other previously reported in the literature. In the metanalysis of Pandolfi et al. a pooled estimate of seronegativity of 9.4% for pregnant women was underlined [24].

The objective of the National Plan for the Elimination of Measles and Congenital Rubella for the period 2010–2015 was the achievement of a percentage of women of childbearing age susceptible to infection that was below 5% [17]. Our data indicate that additional efforts are needed to reach this goal. Until the disease is eliminated in all countries of the world, it will be necessary to maintain high vaccination coverage, and further strengthen the surveillance and investigation of reported cases, ensuring a rapid response to any imported cases.

The new guidelines for physiological pregnancy published in Italy in December 2023 no longer recommend screening for this infection during pregnancy, as it has been declared eliminated [25]. The elimination of the endemic transmission of the rubella virus is described as a major public health success, and the result of tenacious work to achieve high vaccination coverage in the population [25]. However, this claim could lead to a risky underestimation of the problems associated with susceptibility to rubella and the implications related to infection in pregnancy. The collected information from the Italian Behavioral Risk Factor Surveillance System (PASSI), in the period 2017–2020, highlights a significant lack of awareness among women of childbearing age regarding the risks associated with rubella infection during pregnancy. Indeed, a rather high number, nearly 4 out of 10 women (37%), is unaware of their immune status regarding rubella [26]. Pregnancy represents one of the few moments in which women undergo serologic screening, and very often the importance of a preconception visit, aimed at identifying correctable risk factors, before beginning to seek pregnancy is underestimated. Not infrequently, the patient presents to her gynecologist while already pregnant having disregarded the possibility of performing proper counseling and remediating any susceptibility to infection.

It is important to note that the percentage of Italian women susceptible to infection tends to decrease based on parity, probably thanks to a significant strategy to catch up with post-partum women; these data are confirmed by the evidence that women immune to rubella are older than women receptive to rubella. This phenomenon occurs to a lesser extent in non-Italian women, in which 59% of the non-immune group have at least 1 child. These data suggest that compliance to the post-partum vaccination recommendations among these women should have a positive impact. In fact, although Italian women are older than non-Italian women when seropositive for rubella, these data could reflect the demographical characteristic of the pregnant Italian population, which tends to have a first pregnancy at an older age than non-Italian women [27].

A proportion of 63.4% of Italian pregnant women susceptible to rubella in our sample are nulliparous. According to data from the Institute of National Statistics, the fertility rate per woman in Italy is 1.24 [27], so it is plausible that many of these women may not have a second pregnancy.

Both the measles, mumps, and rubella (MMR) and varicella vaccines contain live viruses and are therefore not recommended during pregnancy. As a result, it is important to plan for these vaccinations ahead of time, through preconception counseling if possible. In a study conducted in the United States in 2021, Foley et al. showed how women who did not want to perform rubella vaccination in the preconception phase, despite recommendations, asserted as their motivation the desire not to delay the search for pregnancy [28]. Indeed, opinions from the American Society for Reproductive Medicine and the American College of Obstetricians and Gynecologists uphold that pregnancy is contraindicated for four weeks after vaccination with a live attenuated virus [8]. Shifting the maternal age in the search for first pregnancy and the idea that delaying this search may affect the chances of becoming pregnant may be limiting factors in reaching a threshold of susceptible women that is less than 5%.

It is crucial, therefore, to focus efforts on patients of this population before they experience their first pregnancy. Increasing awareness among healthcare professionals who encounter women in the preconception phase, such as gynecologists or general practitioners, is very important. Rubella testing is offered free of charge to women seeking to conceive, but, evidently, it is still not prescribed frequently enough. This aligns with the fact that the vaccination rate among women of childbearing age is relatively low in Italy, with an average of 44.9% [26].

In this study the rate of vaccinated women is lacking, so it is unknown if the current rubella immunity of the subjects was derived from vaccination or from infections that occurred when rubella was still endemic.

The mandatory vaccination established in 2017 may help future generations but the outcomes of this decision are not yet observable.

Similar strategies to that initiated in Italy in 2017 have been implemented in other parts of the world, yielding significant results that have bought the percentage of women of childbearing age that is susceptible to infection below 5%. In a Korean study conducted from 2004 to 2018, the IgG serum of 72,114 women was analyzed. Women born between 1977 and 1993 benefited from a universal vaccination program initially using only the RCV vaccine and then, later, the MMR vaccine. The overall proportion of seronegative women decreased significantly, from 6.1% in 2004 to 2.5% in 2018. The rate of seronegativity was highest among women who were not targeted for national immunization (born in 1955–1977, 5.2%), while it was lowest among candidates receiving routine and catch-up vaccinations (born in 1986–1993, 2.2%) [29].

## 5. Conclusions

Though rubella endemic infection has been declared eliminated in Europe, it is essential for women of childbearing age to be fully aware of their immune status before embarking on pregnancy and, in case of susceptibility, to take appropriate measures.

The MMR vaccine must be still recommended to all non-immune women and offered to them during preconception and in the post-partum period, as an important element for the prevention of communicable diseases. Vaccination hesitancy can be minimized through a consistent recommendation to all women, one that is offered by medical staff during routine care. Vaccination campaigns and the education of couples about the risks of CRS remain crucial.

## Figures and Tables

**Table 1:** Clinical and demographic characteristics of the sample.

	Seronegative to Rubella (*n* = 713)	Seropositive to Rubella (*n* = 7224)
Maternal characteristics		
Mean age (y), (range)	32.7 (17–46)	34 (14–56)
Nulliparous, *n* (%)	420 (58.9)	3822 (52.9)
1 child, *n* (%)	216 (30.3)	2673 (37)
≥2 children, *n* (%)	77 (10.8)	729 (10.1)

Abbreviation: y (years).

**Table 2:** Clinical and demographic characteristics of the Italian and non-Italian populations.

	Italian Population (*n* = 6512)		Non-Italian Population (*n* = 1425)	
	Seronegative to Rubella (*n* = 574, 8.8%)	Seropositive to Rubella (*n* = 5938, 91.2%)	Seronegative to Rubella (*n* = 139, 9.8%)	Seropositive to Rubella (*n* = 1286, 90.2%)
Maternal characteristics				
Mean age (y), (range)	33.2 (20–46)	34.3 (14–56)	30.5 (17–38)	32.1 (15–55)
Nulliparous, *n* (%)	364 (63.4)	3325 (56)	56 (40.3)	487 (37.9)
1 child, *n* (%)	165 (28.8)	2169 (36.5)	51 (36.7)	508 (39.5)
≥2 children, *n* (%)	45 (7.8)	444 (7.5)	32 (23)	291 (22.6)

Abbreviation: y (years).

**Table 3:** Demographic characteristics (age) of seronegative and seropositive population.

	*n*	Mean Age (y)	Standard Deviation	*p*
Seronegative to rubella	713	32.69	5.6	<0.05
Seropositive to rubella	7224	33.96	5.4	<0.05

Abbreviation: y (years).

**Table 4:** Demographic characteristics (age) of Italian and non-Italian seropositive population.

	*n*	Mean Age (y)	Standard Deviation	*p*
Italian seropositive for rubella	5938	34.36	5.4	<0.05
Non-Italian seropositive for rubella	1286	32.08	5.9	<0.05

Abbreviation: y (years).

## Data Availability

Data supporting these findings are available from the corresponding author upon request.
